# Recent progress in small molecules targeting the acidic tumor microenvironment

**DOI:** 10.1080/14756366.2026.2706109

**Published:** 2026-08-04

**Authors:** Yinuo Fu, Jiahui Song, Chenyang Yu, Yuxi Guo, Bowei Tang, Guangzhong Yang, Yongsheng Zheng, Qiang Wang

**Affiliations:** College of Pharmacy, South-Central Minzu University, Wuhan, Hubei, China

**Keywords:** pH adjustment factors, pH-responsive prodrug, small molecule inhibitors, structure–activity relationship, tumour acidic microenvironment

## Abstract

The acidic tumour microenvironment (pHe 6.5–6.9) is sustained by the Warburg effect and pH regulators, such as monocarboxylate transporters 1 and 4, Na+/H + exchanger 1, vacuolar ATPase, and carbonic anhydrases IX and XII. This environment facilitates tumour invasion, immune evasion, and resistance to therapy in solid tumours. Recent advancements in small molecule inhibitors targeting these pathways have demonstrated potential in molecular design, mechanisms of action, and preclinical studies. However, practical applications face challenges, including metabolic compensation, insufficient target selectivity, and clinical translation difficulties. This article reviews the structural design, structure–activity relationships, biological activity, and clinical trial progress of small molecule inhibitors. It also summarises acid-targeted delivery strategies, such as pH-responsive prodrugs and pHLIP peptides. The aim is to highlight the opportunities and challenges in acid-base regulation within the tumour microenvironment and offer insights for developing a new generation of antitumor drugs with high selectivity and low toxicity.

## GRAPHICAL ABSTRACT

**Figure:**
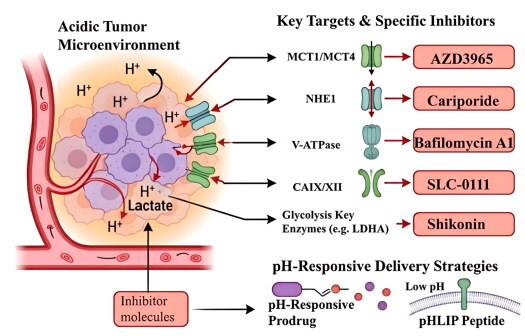

## 1. Introduction

A tumour is not just a cluster of cancer cells; it is a dynamically evolving ecosystem known as the tumour microenvironment (TME). This ecosystem includes various cell types such as cancer cells, fibroblasts, immune cells, and vascular endothelial cells, along with non-cellular components like the extracellular matrix, cytokines, and growth factors^1^. The TME precisely regulates tumour growth, proliferation, invasion, metastasis, and treatment response through direct intercellular contact and paracrine signaling^2^^,^^3^. Its inherent heterogeneity and dynamic evolution pose significant challenges to tumour progression and treatment efficacy^4^. As a result, oncology research has gradually shifted focus from solely targeting cancer cells to intervening in the entire TME, marking a cutting-edge frontier in the development of modern antitumor strategies^5^^,^^6^.

**Figure 1.**
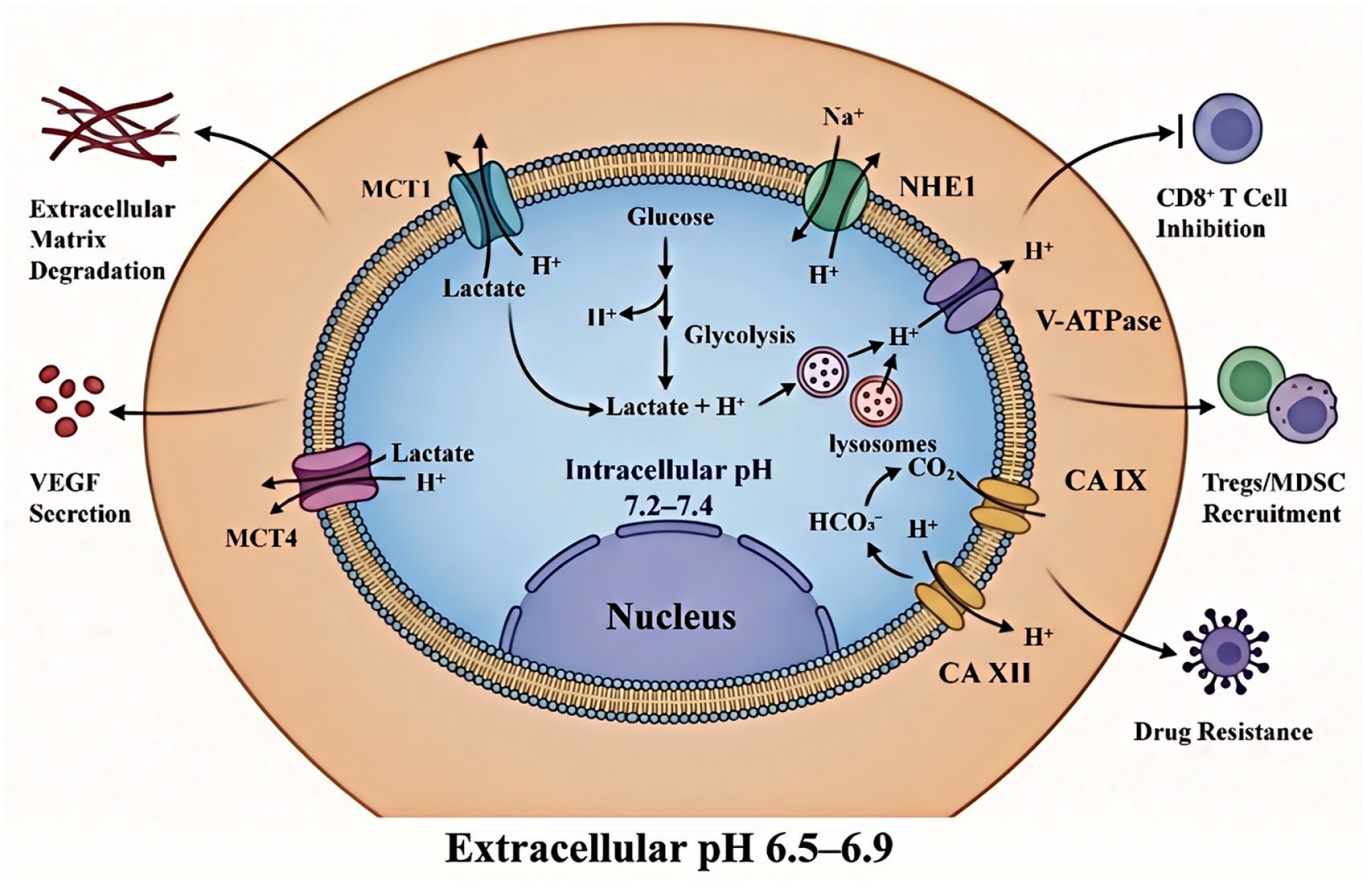
Formation of the acidic tumour microenvironment and its impact on tumours. This figure was designed by the authors using Microsoft PowerPoint 2021 and refined with the AI tool Nano Banana Pro.

A notable feature of the TME is the marked reduction in extracellular pH (pHe ≈ 6.5–6.9), a common trait in nearly all malignant tumours, contrasting sharply with the pHe of approximately 7.4 in normal tissues. This acidic environment results from several synergistic mechanisms. Tumour cells predominantly utilise glycolysis via the Warburg effect, producing substantial lactic acid. This acid, along with protons, is expelled from the cell through monocarboxylate transporters (MCTs). Simultaneously, carbonic anhydrase (CA) facilitates CO_2_ hydration, generating additional protons that further acidify the extracellular space. These processes create an abnormal pH gradient, with relative alkalinity inside tumour cells and significant acidity outside (Figure 1)^7^^,^^8^. The acidic TME is closely linked to another hallmark: hypoxia. Hypoxia, due to abnormal and insufficient tumour vasculature, is common in most solid tumours. This stress induces a metabolic shift towards glycolysis by stabilising hypoxia-inducible factors (HIFs), particularly hypoxia-inducible factor 1α (HIF-1α). HIF-1α upregulates key glycolytic enzymes and glucose transporters, reinforcing the Warburg effect and leading to excess lactic acid production. Additionally, HIF-1α directly induces the expression of pH regulatory proteins like CA IX and MCT4, forming a coordinated response to hypoxia. While tumour acidity was once viewed as a byproduct of hypoxia, it is now recognised as a critical factor profoundly impacting the TME.

The acidic microenvironment of tumours significantly impacts their malignant progression. Invasion and metastasis are facilitated by acidic conditions that activate proteolytic enzymes like cathepsin, which degrade the extracellular matrix, promoting local invasion and distant metastasis. These conditions also induce epithelial-mesenchymal transition (EMT), enhancing tumour cell migration and invasion^9^. In terms of angiogenesis, acidity stimulates tumour and stromal cells to release pro-angiogenic factors, such as vascular endothelial growth factor (VEGF), leading to new blood vessel formation. Acidosis also plays a crucial role in immune evasion by directly inhibiting the cytotoxic functions of effector T cells, including CD8^+^ T cells, and reducing cytokine secretion, such as interferon-γ. It simultaneously promotes the recruitment and maintenance of regulatory T cells (Tregs) and myeloid-derived suppressor cells (MDSCs), establishing an immunosuppressive network. This contributes significantly to the failure or resistance of treatments involving immune checkpoint inhibitors like PD-1/PD-L1 antibodies. Recent studies show that acidosis can enhance IFN-γ-induced PD-L1 expression, further boosting tumour immune evasion^10^. Additionally, the Warburg effect is confirmed as a mechanism for escaping macrophage immune surveillance^11^. Addressing drug resistance, an acidic TME reduces the cell membrane permeability of weakly basic chemotherapy agents through protonation and activates cell survival signalling pathways, decreasing tumour cell sensitivity to radiotherapy-induced DNA damage^12^. Given the critical role of acidic TME in tumour pathophysiology, targeting this acidity has become a promising therapeutic strategy. Current strategies include directacidity neutralisation, acid production inhibition, acid excretion blockage, and intelligent drug delivery systems designed for acidic conditions^13^. Small molecule drugs are particularly promising due to their simple structures, ease of synthesis and modification, effective cell membrane penetration, and ability to target intracellular and extracellular protein molecules^14^^,^^15^. Most pH-regulatory targets, such as CA IX, MCT4, HK2, and LDHA, are transcriptionally controlled by HIF-1α. Thus, targeting tumour acidity is not just about countering a metabolic byproduct; it is a strategic effort to disrupt a coordinated, hypoxia-driven adaptive survival program that supports malignant progression. This perspective supports integrating pH-targeted strategies with broader TME-modulating approaches. This article reviews small molecule inhibitors targeting key glycolytic enzymes and introduces pH regulatory factors like MCTs, sodium-hydrogen exchanger 1 (NHE1), vacuolar ATPase (V-ATPase), and CA IX/XII. It also discusses pH-responsive delivery strategies and examines the challenges and future directions in clinical transformation within this field.

## 2. Small molecule inhibitors targeting the glycolysis–lactate axis

**Figure 2.**
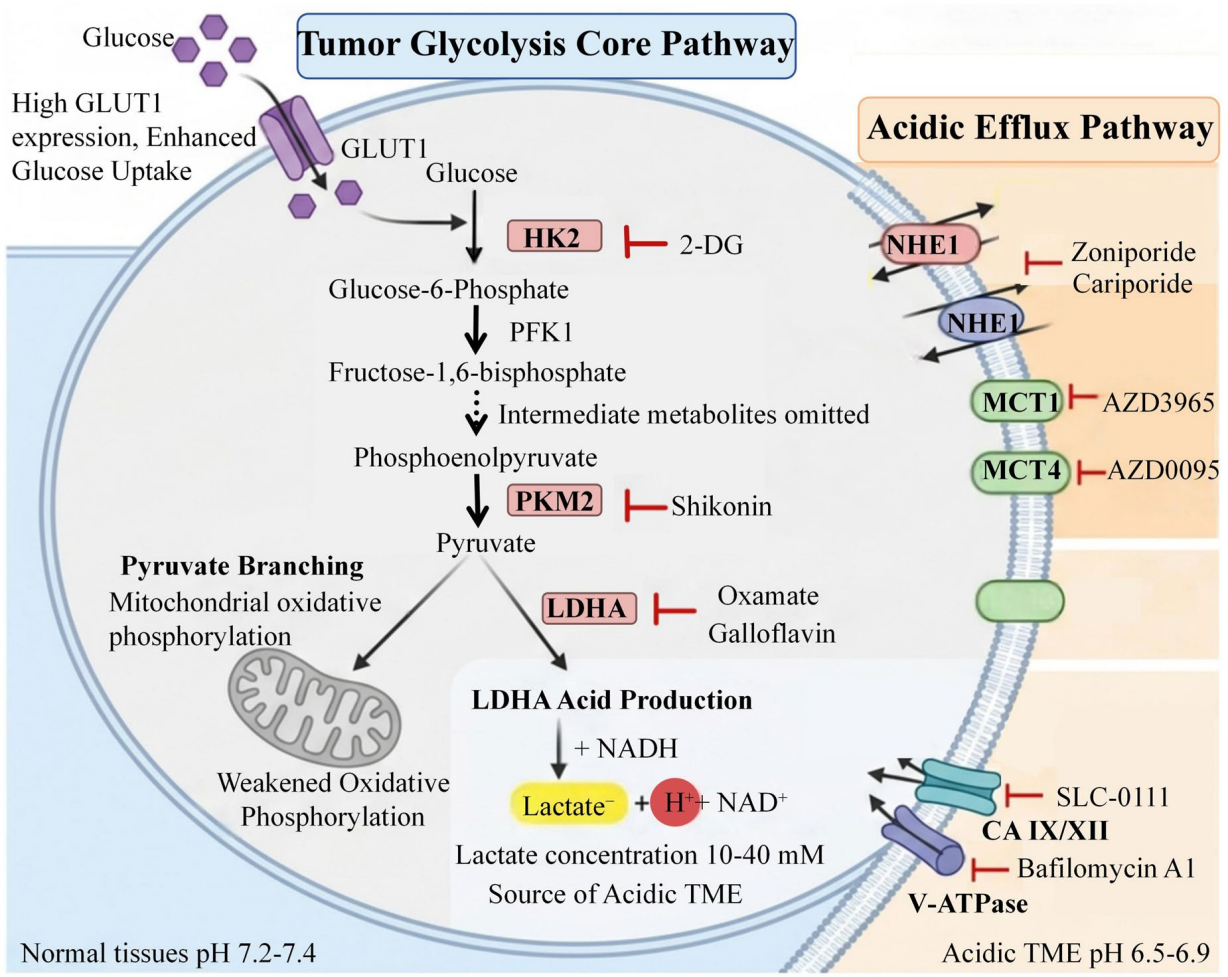
Targets and inhibitors of glycolysis in the acidic TME. This figure was designed by the authors using Microsoft PowerPoint 2021 and refined with the AI tool Nano Banana Pro.

Tumour cells rapidly uptake glucose and produce lactic acid through aerobic glycolysis. This metabolic pathway not only generates energy and biosynthetic precursors but also promotes malignant progression through mechanisms such as lactate modification, immunosuppression, and the induction of EMT^16^. To mitigate these effects, targeting the glycolytic pathway to decrease lactic acid production and inhibit its subsequent efflux has emerged as a pivotal strategy for remodelling the acidic TME (Figure 2). In this section, we review small-molecule inhibitors that sequentially target the glycolysis–lactate axis, beginning with the initial phosphorylation of glucose by hexokinase 2 (HK2), followed by the pyruvate kinase M2 (PKM2)-regulated glycolytic step, lactate generation by lactate dehydrogenase A (LDHA), and ultimately, lactate extrusion via MCT1/4. Collectively, these agents disrupt both the production and efflux of acidic compounds, providing a comprehensive approach to counteracting the protumorigenic effects of tumour acidosis.

### 2.1. LDHA inhibitors

**Figure 3.**
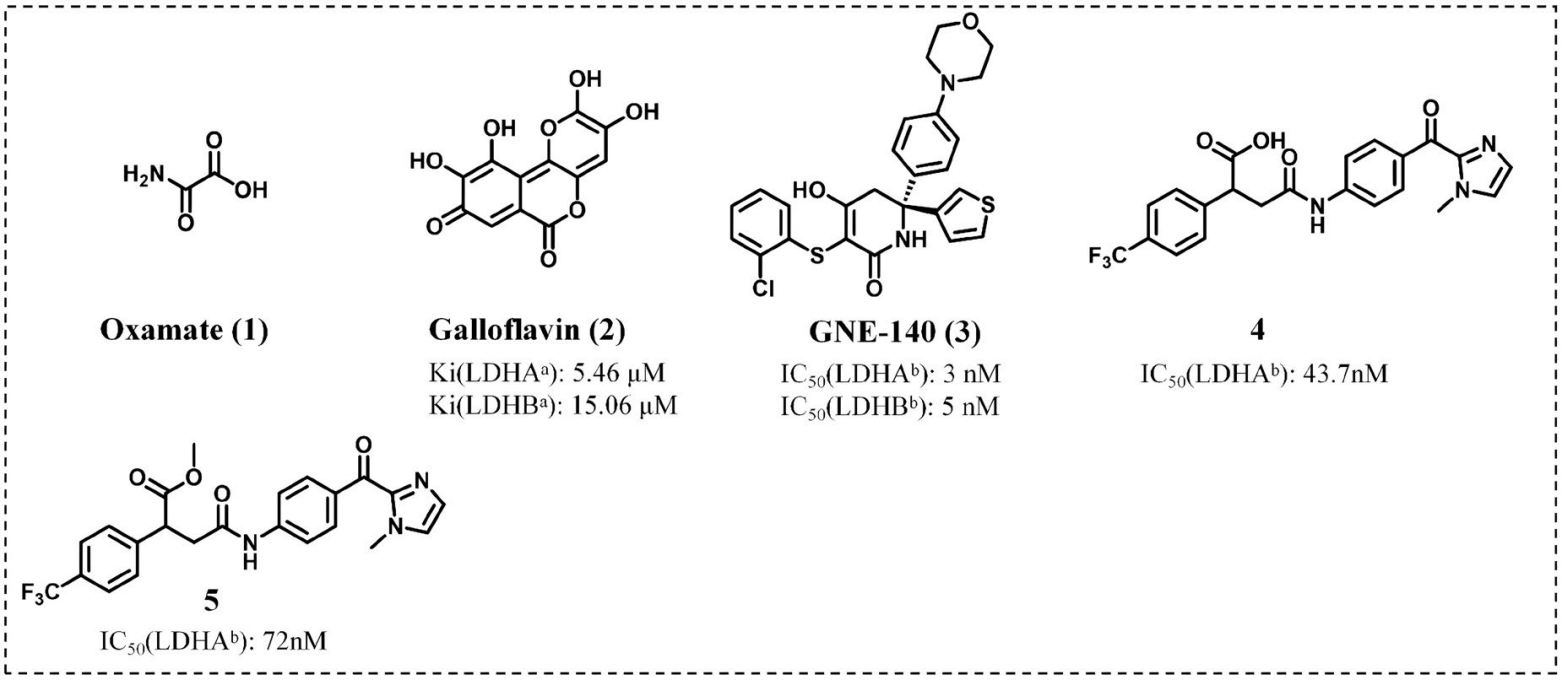
Chemical structures and activities of LDHA inhibitors. ^a^Ki values determined by fluorescence-based enzymatic assay (pH 7.4, 25 °C). ^b^IC50 values determined by enzymatic assay using recombinant human LDHA. This figure was prepared by the authors using KingDraw v3.0 and Microsoft PowerPoint 2021.

LDHA plays a crucial role in glycolysis by converting pyruvate to lactic acid, thus maintaining the acidic TME. Oxamate (Compound 1), a structural analog of pyruvate, competes for LDHA’s active site. It interacts with key residues like Arg168 and Thr247 through hydrogen bonds and salt bridges, effectively blocking lactic acid production (Figure 3)^17^. However, as a first-generation mimetic, oxamate has high polarity (logP < −1) and poor membrane permeability, resulting in sub-millimolar cellular potency. Although it can overcome paclitaxel resistance in preclinical settings, these pharmacokinetic limitations restrict its standalone therapeutic use^18^. Galloflavin (Compound 2), a natural compound, functions as an allosteric inhibitor of LDHA. It significantly reduces the extracellular acidification rate and inhibits proliferation, migration, and invasion in triple-negative breast cancer^19^. The latest generation of inhibitors, such as GNE-140 (Compound 3), demonstrates low-nanomolar inhibition of LDHA, with an IC_50_ of 3 nM, and shows oral bioavailability in mice. The (R)-enantiomer is approximately 18-fold more potent than the (S)-enantiomer, and the compound interacts with a hydrophobic sub-pocket defined by LDHA Gln99, in contrast to LDHB Ala95 ^20^. Despite these advancements, inhibiting LDHA alone leads to metabolic plasticity, causing a shift to mitochondrial oxidative metabolism and fostering drug resistance. No LDHA inhibitor has advanced beyond preclinical evaluation, highlighting the limitations of monotherapy. Therefore, combining LDHA inhibitors with mitochondrial oxidative phosphorylation inhibitors, like metformin, is crucial to fully disrupt the energy compensation mechanism. Additionally, optimising the structure of novel dihydropyrimidine derivatives (specifically compounds 4 and 5) has yielded potent LDHA inhibitors, providing a promising foundation for future drug development^21^.

### 2.2. PKM2 inhibitors

**Figure 4.**
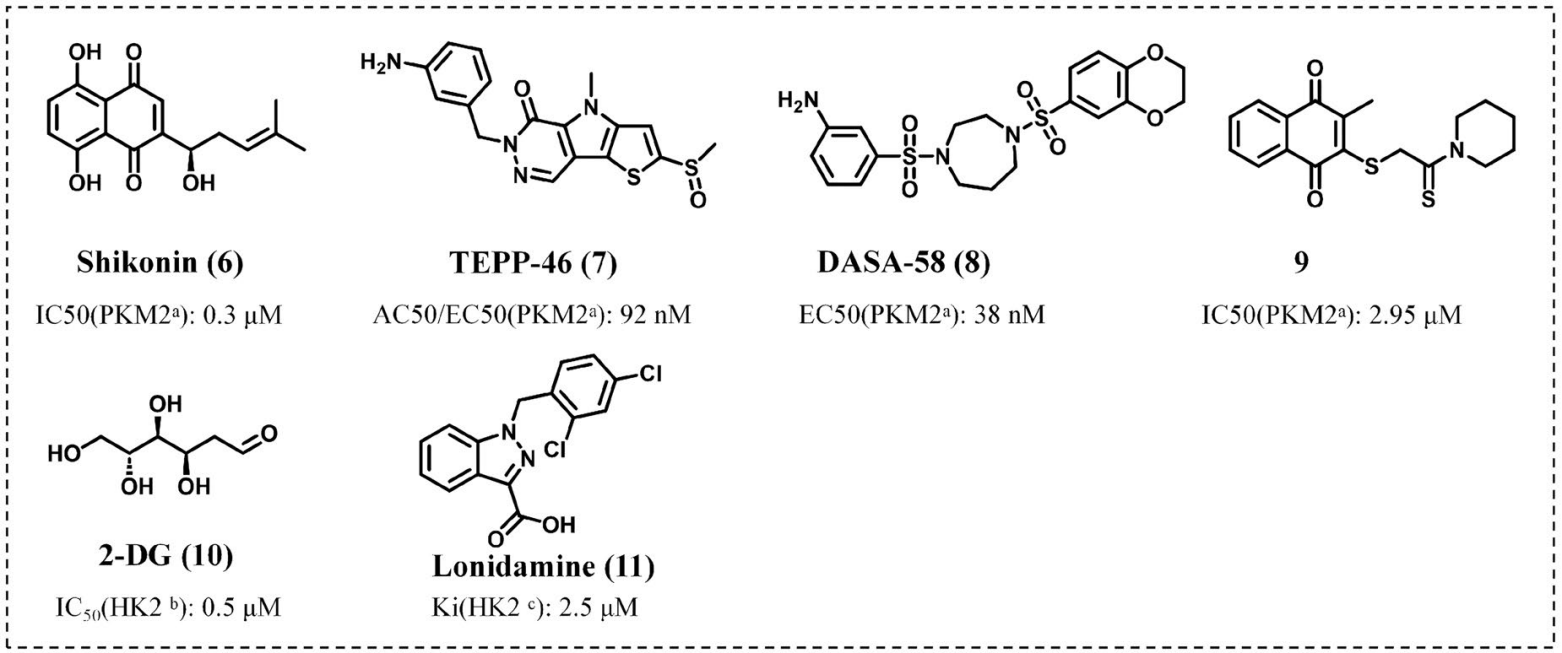
Chemical structures and activities of small molecule modulators targeting PKM2 and HK2. ^a^PKM2 inhibitory activity and PKM2 activating activity were determined by recombinant PKM2 enzyme assay. ^b^The IC_50_ value of 2-DG against HK2 was obtained from a cell viability assay in GIST882 cells. ^c^The *Ki* value of lonidamine against HK2 was determined by HK2 enzyme inhibition assay. This figure was prepared by the authors using KingDraw v3.0 and Microsoft PowerPoint 2021.

PKM2 is a key rate-limiting enzyme in the glycolytic pathway, notable for its dual pro-tumour roles in both metabolic and non-metabolic tumour contexts. In its active tetrameric form, PKM2 catalyses glycolysis, while its low-activity dimer can move to the nucleus, acting as a transcriptional cofactor to promote oncogene expression and EMT, facilitating metastasis^22^. Thus, regulating PKM2’s oligomerisation state has become a promising therapeutic strategy. Shikonin (Compound 6), one of the first identified PKM2 inhibitors, effectively impedes tumour glycolysis and STAT3 phosphorylation, thereby hindering the proliferation and survival of oesophageal squamous cell carcinoma (Figure 4)^23^. Unlike direct inhibition, TEPP-46 (Compound 7) and DASA-58 (Compound 8) act as allosteric “activators” of PKM2. These compounds stabilise PKM2 in its highly active tetrameric state, preventing the formation of low-activity dimers and their nuclear translocation. This action blocks PKM2’s non-metabolic carcinogenic functions while restoring aerobic metabolism and reducing the availability of anabolic intermediates^24^. Additionally, the novel naphthoquinone derivative, Compound 9, developed through structural optimisation, expands the library of tool molecules targeting PKM2^25^.

### 2.3. HK2 inhibitors

HK2 is the primary rate-limiting enzyme in glycolysis, and its abnormal upregulation significantly enhances glucose uptake and phosphorylation in many tumours. 2-Deoxy-d-glucose (2-DG) (Compound 10) is the most widely used inhibitor of HK2, acting as a glucose analogue. When phosphorylated by HK2 to form 2-DG-6-phosphate, it cannot proceed into downstream metabolic pathways, thus competitively inhibiting glucose flux and leading to ATP depletion^26^. 2-DG has entered clinical trials, such as treatment NCT00096707. Although it has an acceptable safety profile, its efficacy as a standalone treatment is limited. Current research is exploring its combination with radiotherapy and chemotherapy to overcome the therapeutic resistance of hypoxic tumour cells. Lonidamine (LND) (Compound 11) is another established glycolytic regulator that inhibits HK2 and disrupts mitochondrial energy metabolism. It also blocks MCT and mitochondrial pyruvate carriers (MPC), causing acidification and energy depletion in tumour cells, thereby enhancing the effectiveness of chemotherapy and radiotherapy^27^^,^^28^. While inhibiting glycolytic enzymes reduces lactate production, tumour cells can maintain an acidic microenvironment through alternative pathways, such as increased lactate export via MCTs. Therefore, blocking lactate extrusion is a complementary and essential strategy to reshape the acidic TME.

### 2.4. MCT1 and MCT4 inhibitors

**Figure 5.**
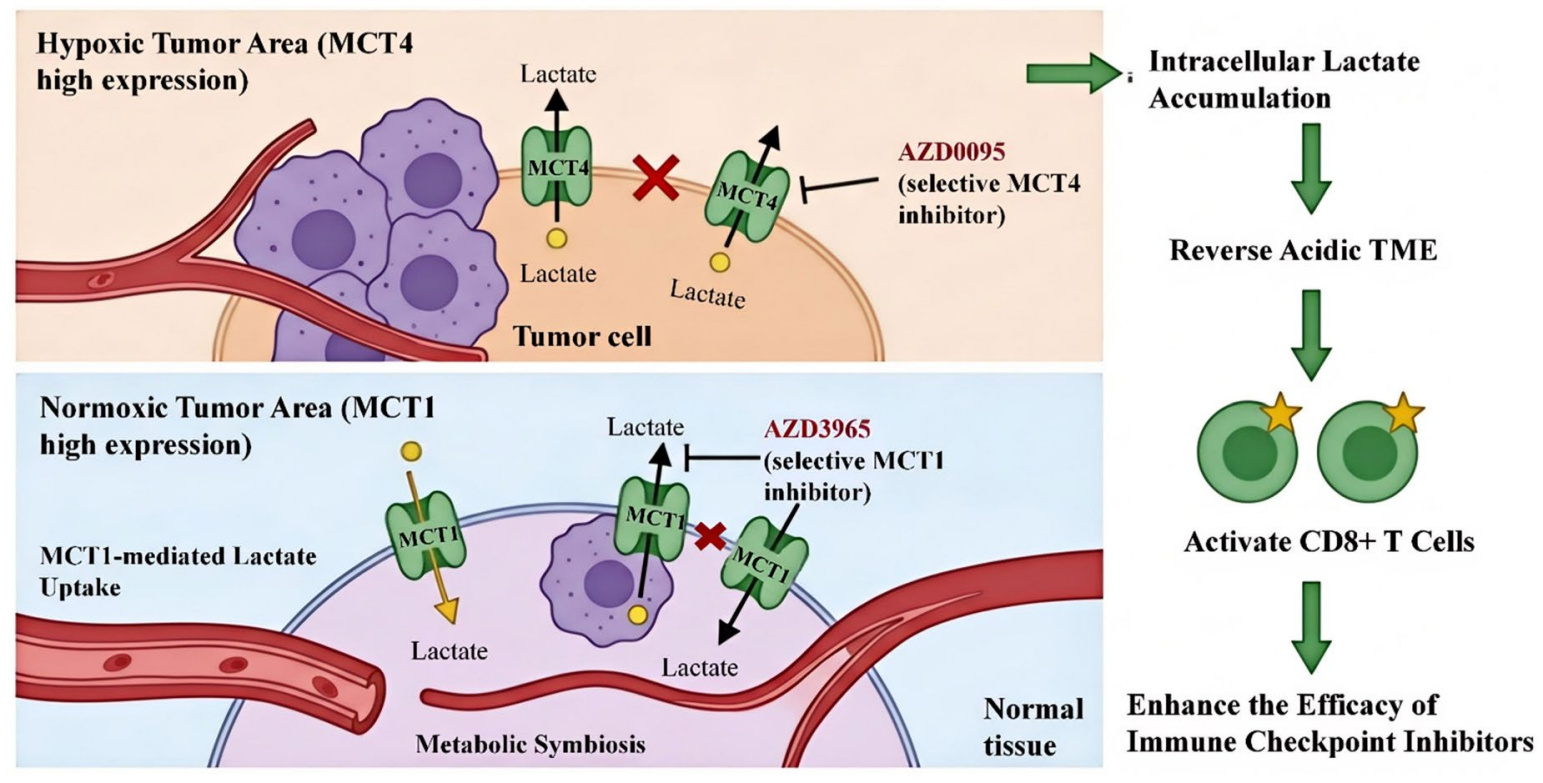
Mechanism of MCT1/4 inhibitors. This figure was designed by the authors using Microsoft PowerPoint 2021 and refined with the AI tool Nano Banana Pro.

Among the monocarboxylic acid transporter family, MCT1 and MCT4 are the primary carriers that regulate lactate-proton co-transport in tumours, influencing the acidic TME^29^. MCT1 is broadly expressed in various tissues and facilitates both the uptake and excretion of lactic acid in normoxic tumour regions. In contrast, MCT4, regulated by HIF-1α transcription, is predominantly expressed in hypoxic tumour areas, where it mainly functions in lactic acid excretion^30^^,^^31^. Both transporters mediate lactic acid shuttling and metabolic symbiosis, contributing to immune evasion, invasion, metastasis, and therapeutic resistance. Consequently, they are key targets for reshaping the TME (Figure 5). Lactic acid production depends on the upstream glycolytic pathway, while MCTs facilitate its excretion from the cell, forming a “production-excretion” metabolic chain. Notably, the endogenous substrate L-lactate has a Km of approximately 3–5 mM for MCT1 and 20–30 mM for MCT4, indicating the lower substrate affinity of the hypoxia-associated MCT4 isoform.

#### 2.4.1. Non-selective MCT inhibitors

**Figure 6.**
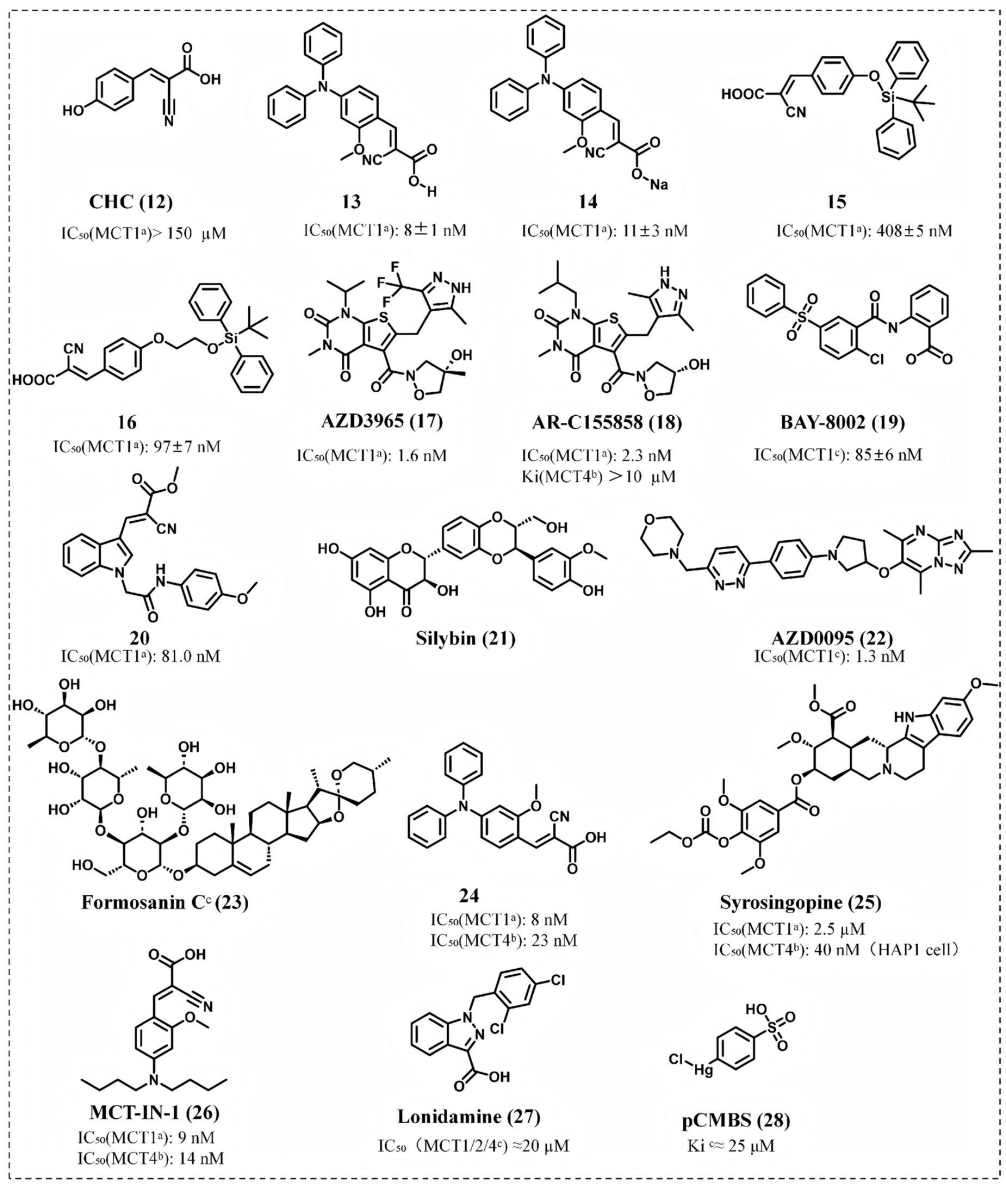
Structures and inhibitory activities of MCT1 and MCT4 inhibitors. ^a^MCT1 IC_50_ values determined by radiolabeled lactate transport assay. ^b^MCT4 IC_50_ or Ki values determined by radiolabeled lactate transport assay (including HAP1 cells for Syrosingopine). ^c^Activity determined by alternative methods: BAY‑8002 (pH‑sensitive fluorescence assay in DLD‑1 cells), AZD0095 (proliferation assay in NCI‑H358 cells), Formosanin C (downregulation of MCT4/CD147), Lonidamine (multi‑transporter inhibition), pCMBS (indirect measurement via CD147 binding, tested in rabbit erythrocytes). This figure was prepared by the authors using KingDraw v3.0 and Microsoft PowerPoint 2021.

Early non-selective MCT inhibitors, like α-cyano-4-hydroxycinnamic acid (CHC) (Compound 12) and phloretin, demonstrated inhibitory constants against MCT1 at the micromolar level, with an IC_50_ of approximately 150 μM (Figure 6). However, these compounds lacked subtype selectivity and exhibited poor in vivo stability, limiting their utility as tool drugs for mechanistic research^32–34^. Recently, researchers have significantly enhanced the activity and pharmacokinetic properties of these compounds through structural optimisation. Gurrapu et al. developed a series of highly potent MCT1 inhibitors by introducing methoxy groups at the ortho position of the benzene ring and *N,N*-dialkyl/aryl groups at the para position, using CHC as the scaffold. Compounds 13 and 14 demonstrated IC_50_ values for MCT1 as low as 8–11 nM, with the sodium salt of compound 14 exhibiting significantly improved water solubility. Structure–activity relationship (SAR) analysis revealed the critical role of the ortho-methoxy group; replacing it with hydrogen reduced activity by over 100-fold, likely due to the loss of a hydrogen bond with Tyr181. The para *N,N*-dimethyl group increases electron density on the benzene ring, enhancing cation-π interactions with Arg306^35^. Furthermore, silicon-based modified cyanocinnamic acid derivatives, such as compounds 15 and 16, designed by Nelson et al., showed significant improvements in metabolic stability while maintaining nanomolar MCT1 inhibitory activity, with IC_50_ values of 408 nM and 97 nM, respectively^36^. Although compounds 13 and 14 exhibit impressive *in vitro* potency, their clinical application is limited by the metabolic instability of the CHC scaffold. The silicon-based isosteric replacement in compounds 15 and 16 offers a rational strategy to overcome this pharmacokinetic limitation while preserving activity.

#### 2.4.2. Selective MCT1 inhibitors

AZD3965 (Compound 17) is an MCT1 inhibitor that has advanced swiftly through clinical trials, completing Phase I studies. This trial, involving patients with advanced solid tumours, established a maximum tolerated dose of 20 mg twice daily. Common adverse events included grade 1–2 nausea, fatigue, and abnormal electroretinograms, with no dose-limiting cardiotoxicity observed. Pharmacokinetic analyses revealed rapid oral absorption and a half-life of approximately 4–6 h. Therapeutic benefits are most notable in tumours with high MCT1 and low MCT4 expression^37^. Despite an acceptable safety profile, AZD3965′s dose-limiting retinal toxicity, as evidenced by abnormal electroretinograms, results from MCT1’s critical role in the retinal pigment epithelium. This leads to a narrow therapeutic index, highlighting the need for selective tumour targeting or the development of isoform-selective inhibitors that spare MCT1 function in vital organs. AR-C155858 (Compound 18) is a highly selective inhibitor that binds to the intracellular transmembrane helix 7–10 region of MCT1, functioning independently of the C-terminal tail. It blocks the conformational changes necessary for substrate transport. This compound also inhibits MCT2 but shows no activity against MCT4^38^. The intracellular binding site on MCT1 helices 7–10 contains residues differing between MCT1 and MCT4, offering a structural basis for its isoform selectivity. BAY-8002 (Compound 19), discovered through high-throughput screening, exhibits heightened sensitivity to hematological malignancies and is useful for PET imaging. However, its efficacy as a monotherapy is limited due to compensatory upregulation of MCT4^39^. Puri et al. synthesised 16 indole compounds, presenting a novel direction for MCT1 inhibitor structures. The optimal compound, designated as 20, exhibited an IC_50_ of 81.0 nM for MCT1 and lacked inhibitory activity against MCT4. This compound selectively induces G2/M phase arrest and apoptosis in MCT1-positive tumour cells and can inhibit the ABCB1 transporter protein, reversing multidrug resistance and sensitising cells to doxorubicin^40^. Although compound 20 is a novel chemotype distinct from classical cyano-cinnamic acid MCT1 inhibitors, its MCT1 potency is weaker than AZD3965, and its interaction with ABCB1 may cause off-target effects. Incomplete SAR data suggest that specific indole substituents are crucial for MCT1 selectivity. Silybin (Compound 21), a natural product, also acts as a selective MCT1 inhibitor, offering a fresh perspective on the origins of MCT1 inhibitors^41^.

#### 2.4.3. Selective MCT4 inhibitors

MCT4 serves as a primary transporter for lactic acid excretion in hypoxic tumour regions and exhibits high specificity for tumours. The inhibition of MCT4 directly leads to lactic acid accumulation and an energy crisis in glycolytic tumour cells^42^^,^^43^. AZD0095 (Compound 22) is a highly selective clinical candidate with an IC_50_ of 1.3 nM, demonstrating over 100-fold selectivity for MCT4 over MCT1, high oral bioavailability, and absence of off-target mitochondrial toxicity. As a monotherapy, it effectively inhibits the proliferation of NCI-H358 cells, which express high levels of MCT4. When combined with the VEGFR inhibitor cedinib, AZD0095 significantly suppresses the growth of transplanted tumours and enhances the tumour immunosuppressive microenvironment^44^. The natural product formosanin C (Compound 23) obstructs lactic acid excretion by downregulating the expression of MCT4 and CD147, thereby inducing tumour cell death^45^.

#### 2.4.4. Direct inhibitors of MCT1/MCT4

Inhibiting a single MCT often leads to compensatory resistance, such as when MCT1 inhibition causes increased MCT4 expression, and vice versa^43^. To address this, simultaneous inhibition of both MCT1 and MCT4 is considered effective in preventing metabolic compensation. N,N-dialkylcyanocinnamic acid derivatives, designed from the CHC skeleton, have achieved dual inhibition at the nanomolar level, with IC_50_ values of 8–48 nM for MCT1 and 11–85 nM for MCT4. Notably, compound 24 targets mitochondria, inhibiting both glycolysis and oxidative phosphorylation. It significantly reduced tumour growth as a standalone treatment in WiDr and MDA-MB-231 tumour models, with enhanced effects when combined with doxorubicin^46^. However, this dual inhibition strategy raises concerns about disrupting lactate homeostasis in normal tissues, particularly affecting the astrocyte–neuron lactate shuttle in the brain and lactate exchange in skeletal muscle. Benjamin et al. discovered that Syrosingopine (Compound 25), a natural product derivative, can inhibit both MCT1 and MCT4, with an IC_50_ of approximately 40 nM for MCT4, making it about 60 times more potent against MCT4 than MCT1. When combined with metformin, it induces a synthetic lethal effect.

#### 2.4.5. Indirect inhibitors of MCT1/MCT4

The drug lonidamine (Compound 27) inhibits both the mitochondrial pyruvate carrier (MPC) and monocarboxylate transporters MCT1, MCT2, and MCT4. At high concentrations, it causes intracellular acidosis by blocking lactic acid efflux^47^. In contrast, the organic mercury compound pCMBS (Compound 28) indirectly disrupts MCT1 and MCT4 by cleaving the disulphide bond of CD147, but it does not affect MCT2. Since MCT1 and MCT4 require the chaperone CD147 (basigin) for surface expression, agents that interfere with this interaction non-selectively impair both transporters^48^. Therapeutic stress can lead to DNA methylation of the MCT1 promoter, forcing tumour cells to rely on MCT4^28^. In such cases, using MCT4 inhibitors can create a synthetic lethal effect. This therapy-induced epigenetic switch, which shifts metabolic dependency from MCT1 to MCT4, is an adaptive resistance mechanism that can make previously sensitive cells resistant to MCT1-selective agents^49^. Additionally, the nuclear receptor pregnane X receptor (PXR) can enhance MCT1 expression transcriptionally, affecting tumour sensitivity to specific kinase inhibitors. This suggests that the PXR–MCT1 axis could serve as a biomarker for combination therapy^50^.

## 3. Small molecule inhibitors targeting non‑glycolytic pH regulation

In contrast to the glycolytic-dependent mechanisms previously described, tumour cells maintain their characteristic inverted pH gradient—marked by intracellular alkalinity and extracellular acidity—through non-lactate pathways. These pathways encompass the electroneutral Na^+^/H^+^ exchange mediated by NHE1, ATP-driven proton pumping facilitated by V-ATPase, and the rapid interconversion of CO_2_ and bicarbonate catalysed by carbonic anhydrases IX and XII. The inhibitors discussed in this section target these independent pH-regulatory networks, thereby offering complementary strategies to mitigate acidosis-driven malignancy without directly influencing glucose metabolism.

### 3.1. NHE1 inhibitors

NHE1 acts as a crucial “proton efflux pump” that maintains a basic internal environment and an acidic external environment in tumour cells^51^. It accomplishes this by exchanging intracellular H^+^ ions for extracellular Na^+^ ions in a 1:1 electrically neutral manner. This process promotes cellular proliferation and inhibits apoptosis while acidifying the extracellular microenvironment, enhancing matrix degradation, and facilitating immune evasion^52^. NHE1 is notably overexpressed in various solid tumours, including breast cancer and glioma. Its activity is regulated by intracellular H^+^ allosteric activation and phosphorylation through oncogenic signalling pathways like MAPK and PI3K/Akt^53^. Inhibiting NHE1 can disrupt proton efflux, leading to intracellular acidification and apoptosis, and can also reduce immunosuppression linked to extracellular acidosis^54^.

**Figure 7.**
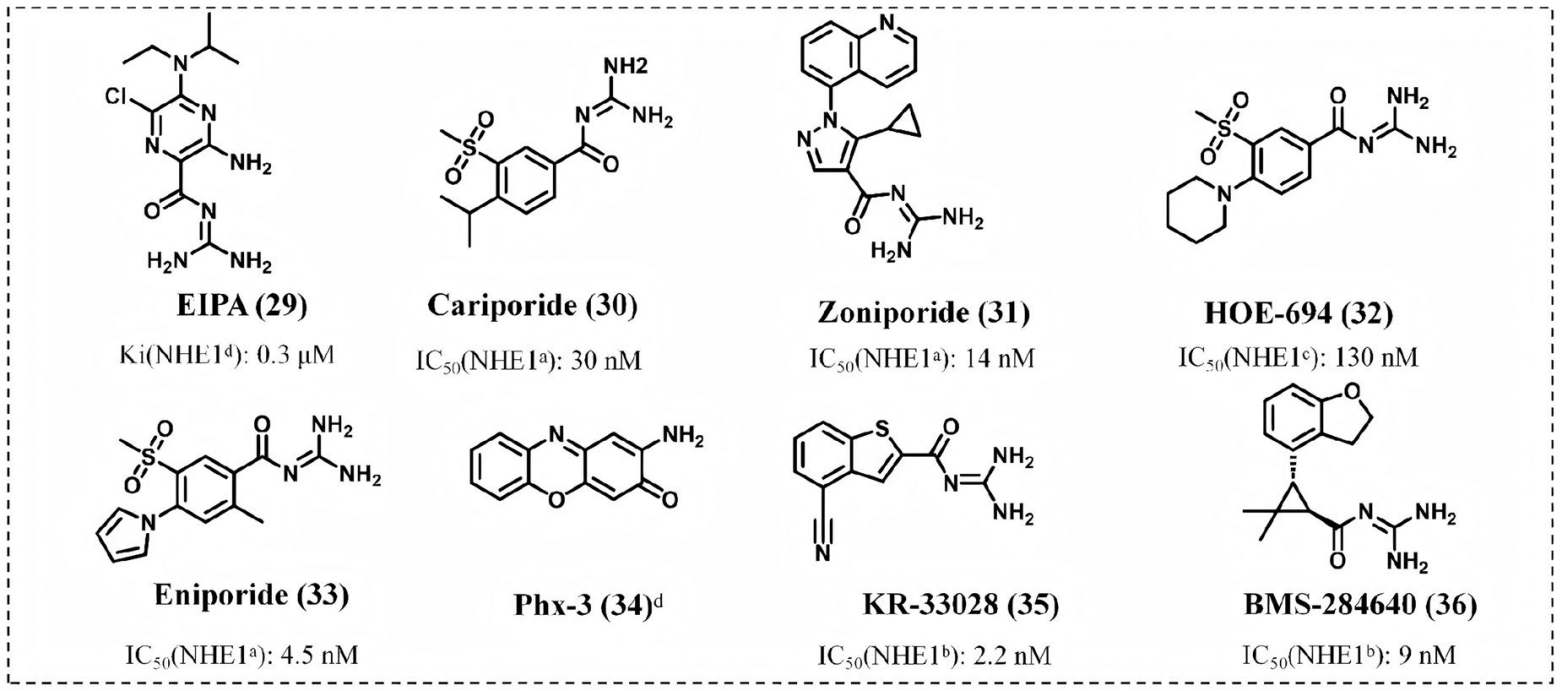
Structures and inhibitory activities of NHE1 inhibitors.^a^ IC_50_ values determined by monitoring intracellular pH changes in CHO‑K1 cells stably expressing human NHE1.^b^ IC_50_ values determined by ^22^Na^+^ uptake inhibition in PS‑120 fibroblasts overexpressing human NHE1.^c^ IC_50_ for HOE‑694 was determined by measuring EIPA‑sensitive ^22^Na⁺ uptake in acidified rabbit erythrocytes.^d^ Ki values were determined by monitoring BCECF-AM fluorescence in Na⁺-loaded cells to measure NHE1‑mediated pH recovery (EIPA). Phx-3 inhibits NHE1 activity, as evidenced by rapid intracellular acidification observed in MKN45/MKN74 gastric cancer cells using BCECF fluorescence. This figure was prepared by the authors using KingDraw v3.0 and Microsoft PowerPoint 2021.

Currently, NHE1 inhibitors are primarily categorised into amiloride derivatives and non-amiloride compounds. Amiloride derivatives feature a pyrazine guanidine core, with 5-(*N*-ethyl-*N*-isopropyl) amiloride (EIPA) (Compound 29) recognised as a classic tool compound (Figure 7)^55^. Screening the amiloride library for dual-targeting uPA/NHE1 identified 6-chloro-5-morpholinyl amiloride (IC_50_ = 129 nM) and 5-(1,4-diazepan-1-yl) amiloride (IC_50_ = 85 nM) as highly selective, low-toxicity probes. Non-amiloride compounds mainly include benzoylguanidine and pyrazole amidine classes. Cariporide (Compound 30) was the first high-selectivity NHE1 inhibitor to enter clinical trials but was terminated due to cardiac toxicity. The EXPEDITION trial (*n* = 5761) on myocardial ischemia–reperfusion showed it reduced infarct size but increased cerebrovascular events and mortality, attributed to cardiac NHE1 inhibition disrupting Na^+^/Ca^2+^ balance, leading to calcium overload. Distinguishing between cardiovascular and oncological contexts is crucial. In the EXPEDITION trial, cariporide was administered acutely at high doses to myocardial ischemia-reperfusion patients, causing fatal calcium overload due to disrupted Na^+^/Ca^2+^ balance. In contrast, proposed oncological applications involve long-term, low-dose administration aimed at modulating tumour pH rather than acute cardiac protection. While this drug repurposing is mechanistically feasible, the narrow therapeutic index remains a major concern. Future cancer trials must carefully optimise dosage and conduct cardiac monitoring. In oncology, cariporide sensitises doxorubicin-resistant breast cancer cells by downregulating NF-κB p65 and MDR1. When combined with doxorubicin, it significantly inhibits resistant tumour growth^56^. However, its monotherapy effect is limited, as prolonged inhibition can upregulate NBCn1, shifting intracellular pH regulation from NHE1 to CO_2_/HCO_3_^-^ alkalisation—a key compensatory resistance pathway^57^. Zoniporide (Compound 31) represents a more advanced class with an IC_50_ of 14 nM and over 200-fold selectivity for NHE2. Its excellent water solubility is due to dual-ionizable acylguanidine and quinoline groups. The cyclopropyl and 5-quinoline groups on the central pyrazole ring restrict C-N bond rotation, reducing conformational entropy and locking the molecule in its bioactive conformation^58^. Despite these improvements, zoniporide’s clinical development stalled, likely due to ongoing cardiac toxicity concerns, indicating that NHE1 selectivity alone may not fully eliminate cardiovascular risks. Other notable NHE1 inhibitors, such as benzoylguanidine HOE-694 (Compound 32) and eniporide (Compound 33), also exhibit nanomolar activity^59^^,^^60^. Compared to the amiloride scaffold, which retains residual activity on epithelial Na^+^ channels and NHE3, non-guanidine and benzoylguanidine chemotypes generally offer better subtype selectivity. Non-guanidine inhibitor Phx-3 (Compound 34) lacks the traditional guanidine group, resulting in lower toxicity; it rapidly acidifies the intracellular environment and induces apoptosis. KR-33028 (Compound 35) selectively targets triple-negative breast cancer, while BMS-284640 (Compound 36) enhances subtype selectivity through its cyclopropane structure^61–63^. Additionally, NHE1 can form a transmembrane metabolic complex with CA IX and MCT1/4, synergistically promoting proton and lactate efflux, providing a basis for dual-target inhibition^64^.

The current strategy involves combining NHE1 inhibitors with immune checkpoint inhibitors to counteract the activation of compensatory pathways often seen with single inhibition. This combination modifies the acidic TME, transforming “cold tumours” into “hot tumours,” enhancing CD8^+^ T cell infiltration, and reversing PD-L1 upregulation. As a result, it significantly enhances the effectiveness of anti-PD-1/PD-L1 therapy, offering a considerable advantage over traditional approaches^65^^,^^66^.

### 3.2. V-ATPase inhibitors

**Figure 8.**
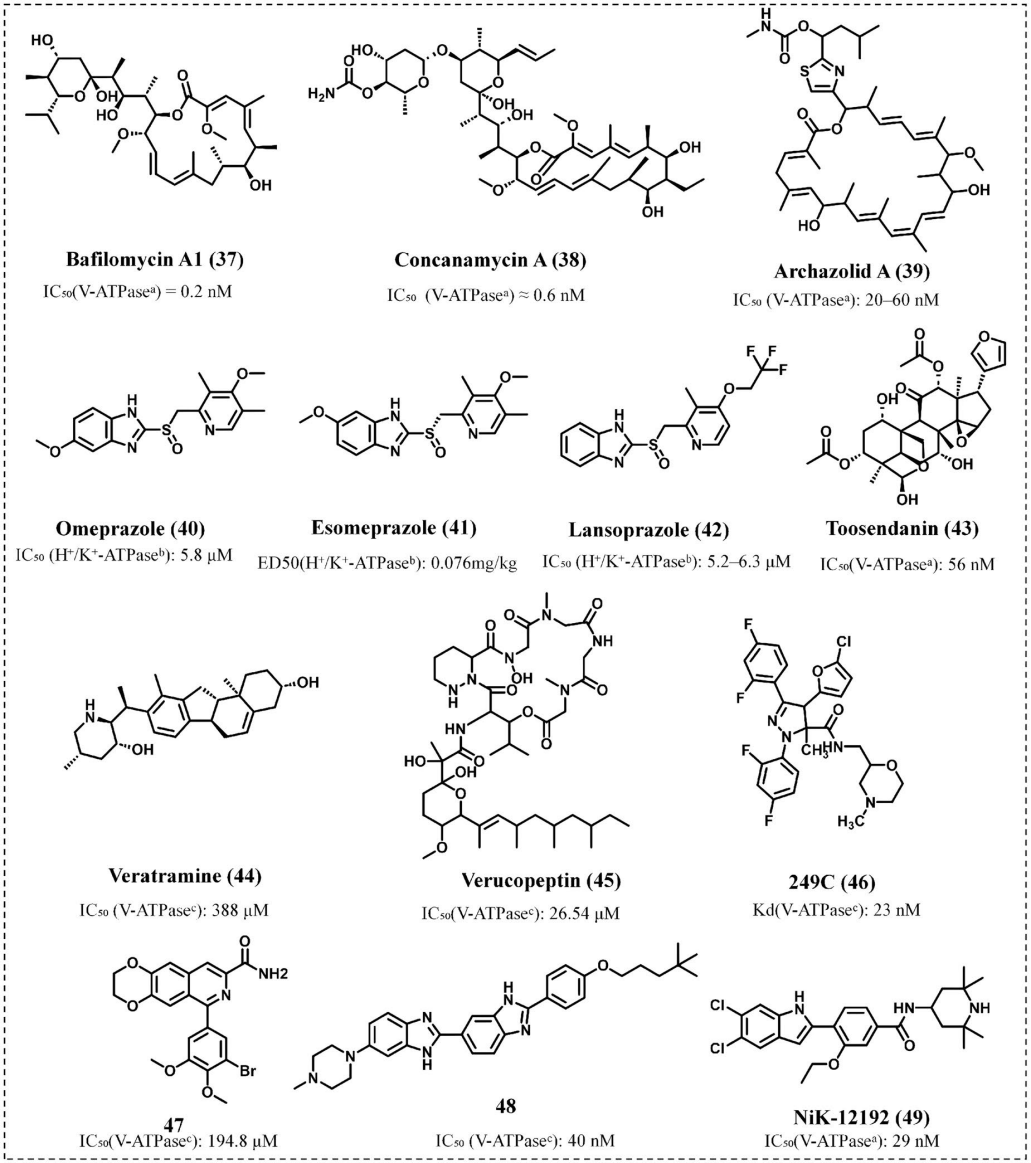
Structures and inhibitory activities of V-ATPase and proton pump inhibitors. ^a^IC_50_ values determined by ATP‑dependent proton transport assay or purified V‑ATPase enzyme activity assay. ^b^IC_50_/ED_50_ values determined by H^+^/K^+^‑ATPase inhibition assay in gastric parietal cells or microsomes. ^c^Activities determined by alternative methods: veratramine (binds ATP6V1C1 in K562R cells), verucopeptin (binds ATP6V1G in HepG2 cells), 249 C (binds ATP6V1H, Kd = 23 nM), 47 (cell viability assay in four cancer cell lines), 48 (cytotoxicity in TNBC cells). This figure was prepared by the authors using KingDraw v3.0 and Microsoft PowerPoint 2021.

NHE1 facilitates electroneutral Na^+^/H^+^ exchange at the plasma membrane, while V-ATPase actively pumps H^+^ using ATP and is found on plasma, lysosomal, and endosomal membranes. Both contribute to extracellular acidification, but V-ATPase uniquely aids lysosomal acidification and autophagic flux. In tumour cells, V-ATPase is overexpressed on plasma and vesicular membranes, extruding H^+^ to maintain intracellular alkalinity, extracellular acidity, and lysosomal function. It is also linked to multidrug resistance^67^^,^^68^. Small-molecule inhibitors targeting V-ATPase can reduce tumour drug resistance and inhibit progression by disrupting pH homeostasis, preventing lysosomal drug sequestration, and reversing ABC transporter-mediated efflux^69^. Classical inhibitors like bafilomycin A1 (Compound 37) and concanamycin A (Compound 38) have been pivotal in understanding V-ATPase’s role in drug resistance (Figure 8). These inhibitors disrupt lysosomal acidification and prevent anthracycline drugs from accumulating in drug-resistant cells, reversing resistance^70^. In primary acute myeloid leukaemia (AML) cells, they show dose-dependent anti-proliferative and pro-apoptotic effects^71^. However, their nanomolar cytotoxicity across cell types limits systemic use due to V‑ATPase’s essential roles in renal acid-base balance, bone resorption, and synaptic vesicle loading. Archazolid A (Compound 39), a selective V-ATPase inhibitor, interferes with E-cadherin turnover, inhibits EMT, and suppresses tumour stem cell phenotypes, showing potent effects in leukaemia and solid tumors^72^^,^^73^. The repurposing of old drugs and natural product inhibitors has expanded therapeutic options. Proton pump inhibitors (PPIs) like omeprazole (Compound 40), esomeprazole (Compound 41), and lansoprazole (Compound 42), although not directly binding to V-ATPase, elevate intracellular and lysosomal pH, diminishing the proton gradient and reversing drug resistance. Lansoprazole can bind to ABCB1/ABCG2 and obstruct lysosomal storage, aiding in reversing multidrug resistance^69^^,^^74^. Their safety and oral bioavailability make them suitable for combination therapy, but their clinical use is limited by weak V‑ATPase inhibition, requiring high doses that may cause off-target effects. Lansoprazole’s interaction with ABCB1/ABCG2 also raises concerns about drug interactions. The natural triterpene Toosendanin (Compound 43) directly inhibits V-ATPase, raises lysosomal pH, hinders chemotherapy-induced autophagy, and enhances chemotherapy efficacy against tumors^75^^,^^76^. The alkaloid Veratramine (Compound 44) binds to the V-ATPase subunit ATP6V1C1, obstructing proton pump activity and disrupting the autophagy-lysosomal pathway crucial for tumour survival^77^. New inhibitors targeting specific subunits are emerging. Verucopeptin (Compound 45) binds to the ATP6V1G subunit, inhibiting V-ATPase and mTORC1 signalling, showing high potency and low toxicity against multidrug-resistant tumors^78^. Compound 249 C (Compound 46) selectively binds to V-ATPase, preventing lysosomal acidification and inhibiting RAS-driven autophagy and macropinocytosis in tumours. It suppresses tumours in kRAS-mutated lung and colon cancer models and is in phase I clinical trials (NCT04678648)^79^. However, the high sequence conservation of V‑ATPase subunits across tissues means subunit-selective agents face a narrow therapeutic index. The dual-target inhibitor Compound 47 inhibits both tubulin and V-ATPase, triggering

immunogenic cell death and enhancing tumour immune infiltration, offering a new avenue for combined immunotherapy^80^. Additionally, the novel bisbenzimidazole derivative Compound 48 is a unique V-ATPase inhibitor with an IC_50_ of 15 nM for triple-negative breast cancer and minimal toxicity to normal cells^81^. Nik-12192 (Compound 49) reduces lysosomal acidity, alters integrin distribution, and induces nest-loss apoptosis by inhibiting V-ATPase^82^. The dual role of V‑ATPase in plasma membrane and lysosomal pH regulation complicates pharmacodynamic assessment. It is often unclear if anti-tumour effects result from disrupted extracellular acidification, impaired lysosomal degradation, blocked vesicular drug sequestration, or a combination, complicating biomarker development and rational combination design.

The alkalisation strategy, designed to neutralise the acidic TME, does not directly target V-ATPase. Instead, it indirectly modulates tumour pH through a buffering system and serves as an adjunct to other therapies. Daily oral administration of potassium citrate has been shown to elevate bicarbonate levels in the bloodstream, neutralising the extracellular pH of tumours in mouse models and significantly enhancing the efficacy of TS-1 in pancreatic cancer. Similarly, sodium bicarbonate therapy selectively increased pHe and inhibited spontaneous metastasis in breast and prostate cancer models. This effect is associated with the inhibition of cathepsin B activation^83^^,^^84^.

### 3.3. CAIX/CAXII inhibitors

Carbonic anhydrases are zinc-containing metalloenzymes that catalyse the reversible hydration of CO_2_, producing HCO_3_^-^ and H^+^. The tumour-associated isoforms, CA IX and CA XII, are highly expressed in various solid tumours. They maintain an intracellular alkaline and extracellular acidic pH gradient, which promotes proliferation, invasion, metastasis, drug resistance, and immune evasion^85^^,^^86^. CA IX has a catalytic turnover (kcat) of approximately 3.8 × 10^5^ s^−1^ with a Km of about 4.5 mM for CO_2_, while the ubiquitous cytosolic CA II reaches a rate of ∼1.1 × 10^6^ s^−1^, making it one of the fastest known enzymes. Although optimised sulphonamide inhibitors achieve low-nanomolar to sub-nanomolar Ki values, the exceptional catalytic efficiency of carbonic anhydrases requires nearly complete inhibition (>99%) for a significant physiological impact. This threshold is challenging to maintain consistently in the TME.

#### 3.3.1. Sulphonamide inhibitors

**Figure 9.**
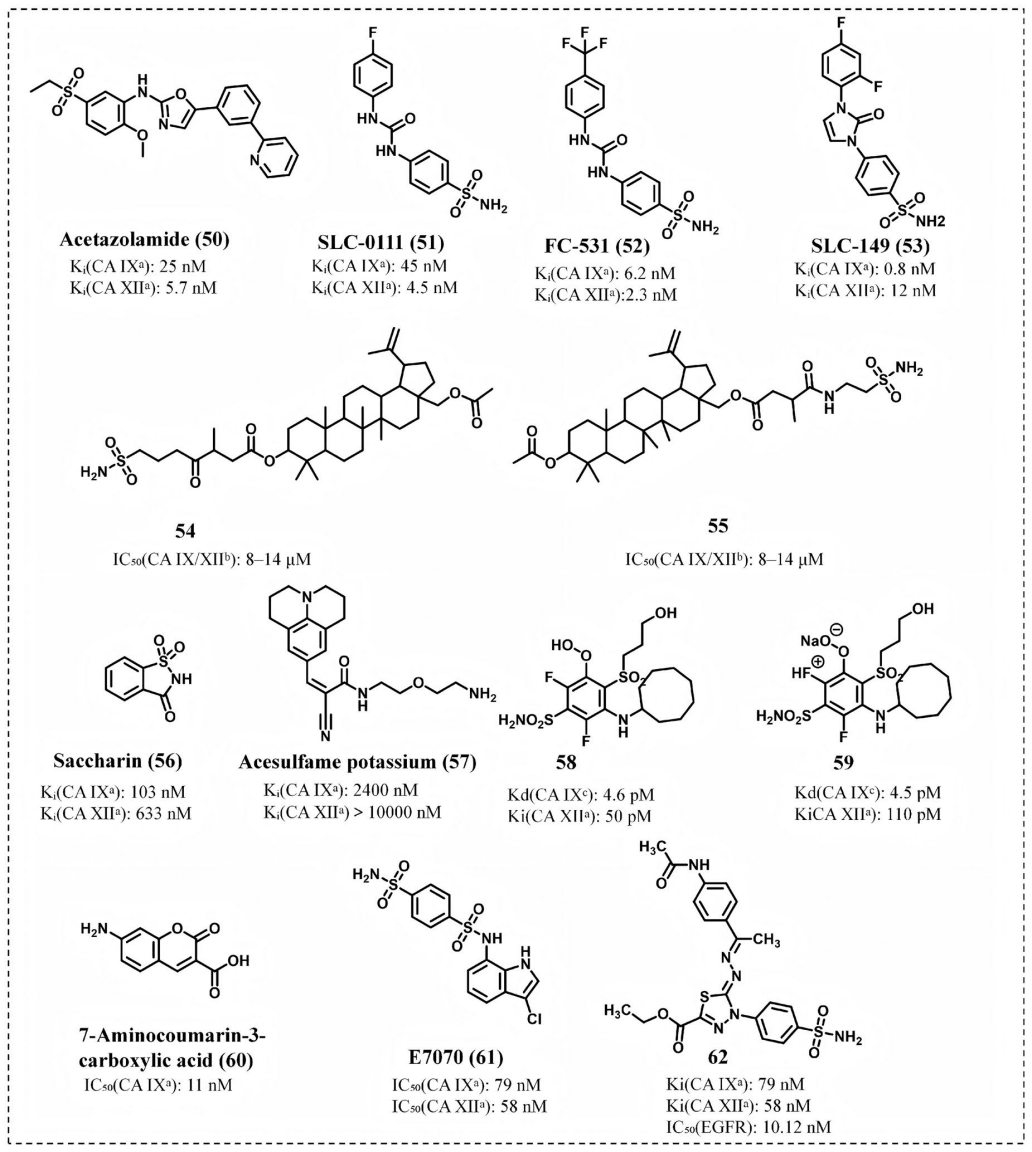
Structures and inhibitory activities of carbonic anhydrase IX/XII inhibitors. ^a^Ki/IC_50_ values for CA IX and CA XII were determined by a stopped-flow CO_2_ hydration assay using recombinant human CA isozymes. ^b^IC_50_ values of betulin sulphonamides^54^^,^^55^ were determined by a cell viability assay (MTT) in breast cancer cell lines (MCF-7, T47D, SKBR3, HS578T, MDA-MB-231, BT-20). ^c^*K*_d_ values of fluorinated benzenesulfonamides^58^^,^^59^ were determined by a thermal shift assay using recombinant CA IX/XII. This figure was prepared by the authors using KingDraw v3.0 and Microsoft PowerPoint 2021.

Sulphonamide inhibitors are widely studied and structurally modified CA inhibitors. They typically work by reversibly coordinating the nitrogen atom in the sulphonamide group (–SO_2_NH_2_) with the Zn^2+^ ion at the CA active site, thus inhibiting the enzyme’s catalytic function. Early compounds, such as acetazolamide (AAZ) (Compound 50), showed poor subtype selectivity. Recent research has focused on achieving selective inhibition of CA IX/XII by optimising tail substituents (Figure 9)^87^. This approach uses the universal zinc-binding pharmacophore while customising substituents to fit the unique active-site architecture of CA IX, particularly the hydrophobic pocket formed by Val131 (as opposed to the bulkier Phe131 in CA II) and residue 67 (Gln in CA IX vs. Asn in CA II). SLC-0111 (Compound 51) is a leading clinical candidate, with Ki values of 45 nM for CA IX and 4.5 nM for CA XII^88^. In a Phase I clinical trial (NCT02215850), daily oral administration over 28 days demonstrated good safety, establishing the recommended Phase II dose (RP2D) at 1000 mg/day^88^. Preclinical studies have shown that SLC-0111 can enhance the effectiveness of various drugs, such as dacarbazine, temozolomide, doxorubicin, and 5-fluorouracil, and works synergistically with radiotherapy to inhibit glioblastoma^89^^,^^90^. However, as a monotherapy, sustained CA IX inhibition results in limited objective responses, indicating that CA IX primarily aids tumour survival under hypoxia rather than directly promoting proliferation. This suggests that CA IX inhibitors are mainly useful as chemosensitizing and radiosensitizing agents^88^. Additionally, CA XII co-expression may compensate for CA IX inhibition, as both isoforms catalyse CO_2_ hydration and help maintain pH gradients^91^. Its analogue, FC-531 (Compound 52), has a Ki value of 6.2 nM for CA IX and a Ki value of 2.3 nM for CA XII, and its tumour suppression effect is comparable^9^,^0^. Another related compound, SLC-149 (Compound 53), is a rigid cyclic ureido benzenesulfonamide derivative structurally optimised from the linear scaffold of SLC-0111. It exhibits remarkably potent isozyme inhibitory activity, with Ki values of 0.8 nM and 12 nM for CA IX and CA XII, respectively^92^. Betulinic acid (BA) sulphonamide derivatives, such as 13b (Compound 54) and 15b (Compound 55), represent optimised natural products with CA IX inhibition and anti-tumour properties. These derivatives bind to Zn^2+^ via sulphonamide groups, inhibit CA IX expression and activity, reduce extracellular acidity, and suppress HIF-1α. They also decrease tumour stem cell clone formation, enhance sensitivity to radiotherapy and chemotherapy, and show significant efficacy in treating triple-negative breast cancer^93^. Saccharin (SAC) (Compound 56) acts as a selective CA IX inhibitor with a Ki of 103 nM, achieving selectivity due to differing residues at positions Gln67 and Asn67. Potassium acesulfame (ACE) (Compound 57), another artificial sweetener, also selectively inhibits CA IX. The BTD series derivatives, synthesised with ACE as the lead compound, show enhanced activity; for example, the Ki of 7-fluorine BTD against CA IX is 19.1 nM^94^. Significant progress has been made with fluorinated dimethyl-substituted benzenesulfonamides. Compounds 58 and 59 exhibit a Kd of approximately 4.5 pM for CA IX, with selectivity over CA II exceeding 1000-fold^95^. X-ray co-crystal structures reveal a distinct binding mode: the 3-cyclooctylamino substituent occupies the hydrophobic active-site pocket unique to CA IX, while the 5-methoxy/ethoxy group extends into a hydrophilic region. This rational design has produced the most potent and selective CA IX inhibitors reported to date. However, the gap between biochemical potency and *in vivo* efficacy likely stems from pharmacokinetic challenges in maintaining sustained, high-concentration target engagement in poorly perfused hypoxic tumour compartments^95^.

#### 3.3.2. Non-sulphonamide inhibitors

Non-sulphonamide CA inhibitors offer innovative mechanisms that do not rely on zinc ion binding, facilitating the development of highly selective CA IX inhibitors. Among these, coumarin derivatives are particularly notable. Instead of coordinating with zinc, they induce water dissociation, forming an active trans conformation that blocks the CA active-site entrance, acting as time-dependent suicidal substrates^96^. This mechanism provides a selectivity advantage, as the coumarin binding pocket at the active-site rim varies more across isoforms than the zinc coordination sphere. However, the irreversible, time-dependent nature complicates pharmacokinetic/pharmacodynamic (PK/PD) correlations and raises concerns about prolonged target engagement beyond the therapeutic window. Building on this scaffold, numerous nanomolar-potent CA IX inhibitors have been synthesised. Notably, a group of 7ACC (7-aminocarboxycoumarin) derivatives (Compound 60), despite retaining the 3-carboxyl group, primarily impede lactic acid uptake by targeting monocarboxylate transporters (MCTs) rather than CA. This results in growth inhibition in MCT-overexpressing cells (SiHa, HCT116, MCF-7), highlighting off-target complexities in this class^97^. Additionally, thiazolidinone, triazole, and other heterocyclic compounds have been designed as CA IX inhibitors. For instance, E7070 (Compound 61), a clinical-stage antitumor agent, exhibits dual activity: it induces RBM39 degradation and independently inhibits CA IX and CA XII with IC_50_ values of 79 nM and 58 nM, respectively. It also enhances glioblastoma sensitivity to radiotherapy and temozolomide by disrupting pH homeostasis^98^^,^^99^. Significant advancements have been achieved with dual-target inhibitors. A sulfonamide phenyl-dihydrothiadiazole derivative (Compound 62) not only retains inhibitory activities against CA IX and CA XII but also demonstrates potent epidermal growth factor receptor (EGFR) inhibitory activity (IC_50_ = 5.92 nM). This compound exhibits superior antitumor effects *in vivo* compared to monotherapy^100^. Furthermore, combining CA IX inhibitors with radiotherapy, chemotherapy, and mTOR inhibitors has shown a synergistic effect in preclinical studies^99^. The innovative strategy of integrin-facilitated lysosomal degradation (IFLD) has led to the development of the bifunctional compound Sul-L1-RGD. This compound connects a high-affinity CA IX ligand with a cyclic RGD peptide, allowing it to bind simultaneously to integrins and CA IX on tumour membranes. This dual binding triggers endocytosis and subsequent lysosomal degradation of the complex. When treated with 5 nM of Sul-L1-RGD for 8 h, more than 50% of CA IX was degraded, achieving hypoxic tumour suppression levels comparable to SLC-0111. Although this catalytic removal strategy partially addresses the need for sustained target occupancy, it presents translational challenges. These include the potential immunogenicity of the RGD moiety, competition with endogenous integrin ligands, and the necessity for coordinated engagement of two cell-surface receptors—integrin αvβ3/αvβ5 and CA IX—which may have varying expression levels across different tumour subtypes^101^.

## 4. pH-responsive small molecule prodrugs

**Figure 10.**
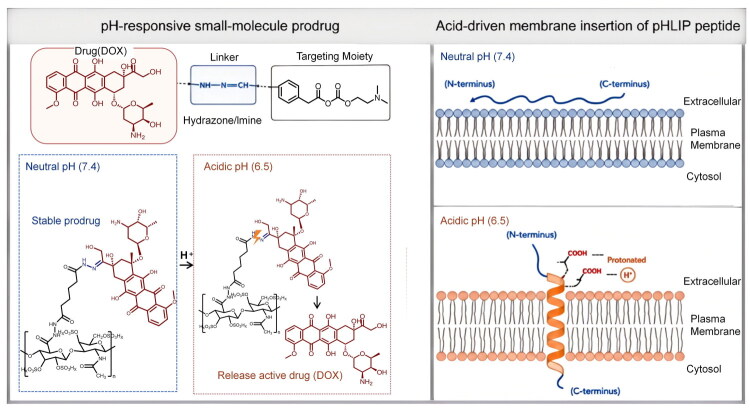
Acidic microenvironment targeting mechanisms of pH-responsive small molecule prodrugs and pHLIP peptides.This figure was designed by the authors using Microsoft PowerPoint 2021 and refined with the AI tool Nano Banana Pro.

Instead of directly inhibiting pH-regulating proteins, an alternative strategy leverages the acidic TME as a natural trigger for targeted drug delivery. pH-responsive prodrugs and pHLIP peptides can specifically release or anchor therapeutic agents within acidic tumours, reducing systemic toxicity. This section reviews recent progress in acid-labile linkers and pHLIP-based targeting (Figure 10).

### 4.1. Prodrug design based on acid-sensitive linker bonds

A pH-responsive prodrug integrates acid-labile chemical linkages into the drug compound to ensure stability and inertness in the bloodstream at physiological pH. Once in the acidic TME or after tumour cell internalisation into endosomes or lysosomes, these linkages cleave, releasing the active parent drug. Common acid-labile bonds include hydrazone, imine, and ester bonds^102–104^. Acylhydrazone bonds are widely used due to their stability at physiological pH and rapid hydrolysis in acidic environments. This study developed a trifunctional acylhydrazone linker that can simultaneously carry ketone-based drugs, like doxorubicin, and nucleophilic fluorophores. When triggered by acidic conditions, the two payloads are released in sequence, providing a chemical basis for spatio-temporal coordinated dual release^105^. The hyaluronic acid (HA) - hydrazone bond - doxorubicin (DOX) prodrug micelle (HAD) system integrates active targeting with cascade activation. HA targets the CD44 receptor, promoting the cleavage of the hydrazone bond in the acidic TME and lysosomes. This process is enhanced by enzymatic hydrolysis, ensuring high selectivity away from normal cells^106^. Additionally, a pH-responsive dual-loading strategy was used to prepare nanoparticles (DNCaNPs) via a CaCO_3_-assisted dual emulsion method, co-loading DOX and the indoleamine 2,3-dioxygenase 1 (IDO1) inhibitor NLG919^107^.

### 4.2. pHLIP peptide

Unlike acid-sensitive chemical bonds, the pH low insertion peptide (pHLIP) targets acidic cell surface regions through a unique physical insertion mechanism^108^^,^^109^. At a physiological pH of 7.4, pHLIP adopts an irregularly coiled shape and weakly attaches to the cell membrane. When it encounters an acidic environment (pH ≈ 6.5), the neutralisation of aspartic and glutamic acid residues increases its hydrophobicity. This change causes pHLIP to spontaneously fold into a transmembrane α-helix, inserting its C-terminal into the cytoplasm while keeping the N-terminal outside the cell. This mechanism enables pHLIP to effectively anchor within the acidic environments of tumours.

The conjugation of exogenous haemagglutinin peptide antigen with pHLIP effectively targeted and anchored onto the acidic surfaces of tumour cells in a pre-immunized mouse model. After intravenous administration of HA-PHLIP, the construct successfully localised to the acidic tumour cell surface, allowing recognition by circulating anti-HA antibodies. This process significantly inhibited the growth of “cold tumours,” such as triple-negative breast cancer and MHC-I negative melanoma, through mechanisms like antibody-dependent cell-mediated cytotoxicity (ADCC)^108^. Functionalising mesoporous organosilicon nanoparticles with pHLIP enables precise targeting of acidic microenvironments. Cellular uptake at pH 6.5 was significantly higher than at pH 7.4, leading to a twofold increase in tumour drug enrichment in vivo. Moreover, the tumour suppression effect exceeded that of free DOX and the unmodified group^110^. pHLIP technology is also applicable for targeting other critical cells within the acidic TME. Tumour-associated macrophages (TAMs), which rely heavily on glycolysis during M2 polarisation, exhibit pronounced cell surface acidity. pHLIP efficiently targets and activates macrophages, including CD206^+^ TAMs, demonstrating specific labelling of TAMs in lipopolysaccharide-induced pneumonia lesions and CT26 tumours, with minimal targeting of other immune cells^109^. This advancement significantly reshapes the tumour immune microenvironment.

## 5. Summary and outlook

Focusing on the acidic tumour microenvironment offers a novel strategy for tackling the invasion, immune evasion, and multidrug resistance of solid tumours. Significant progress has been made in developing small molecule inhibitors targeting pH regulatory elements such as MCTs, CA IX/XII, NHE1, and V-ATPase. Several promising drugs have advanced to clinical trials^111–114^. However, challenges remain, including overlapping metabolic pathways, insufficient target specificity, toxicity to normal tissues, and tumour heterogeneity, which hinder clinical translation^115^^,^^116^. Notably, many pH-regulatory targets, including CA IX, MCT4, and glycolytic enzymes, are transcriptionally regulated by HIF-1α. Therefore, future therapeutic strategies should consider the hypoxia–acidity signalling axis as a combined target, rather than treating tumour pH in isolation from the hypoxic conditions that drive its dysregulation. Each class of inhibitors encounters specific limitations. Glycolytic enzyme blockers (LDHA/PKM2/HK2) decrease lactate production but can easily induce a metabolic shift towards oxidative phosphorylation. MCT inhibitors directly inhibit lactate efflux and disrupt metabolic symbiosis; however, blockade of a single isoform leads to compensatory upregulation of the corresponding transporter. NHE1 inhibitors reverse the pH gradient and provide dual antitumor and immunomodulatory effects, yet their application is limited by cardiovascular risks. V-ATPase inhibitors influence both extracellular acidification and lysosomal homeostasis, presenting broad anti-resistance activity, although they exhibit poor tissue selectivity. CA IX/XII inhibitors specifically target hypoxia-induced acidification and function as effective chemo/radiosensitizers, yet their antiproliferative efficacy as monotherapy remains inadequate.

A major challenge in targeting tumours is overcoming compensatory drug resistance driven by metabolic plasticity^117^. Tumour cells maintain pH homeostasis and energy metabolism by upregulating alternative pathways, such as LDHA, MCT1/4, NHE1, or CAs^33^^,^^65^. For example, inhibiting MCT1 leads to increased MCT4 expression, and vice versa^43^. Similarly, prolonged NHE1 inhibition results in the upregulation of NBCn1, shifting the mechanism from Na^+^/H^+^ exchange to bicarbonate-dependent alkalization^57^. When LDHA or glycolysis is inhibited, tumour cells switch to mitochondrial oxidative phosphorylation, using fatty acids or glutamine for energy^118^. Therapeutic stress can also methylate the MCT1 promoter, making tumours dependent on MCT4 for survival^51^. Given these compensatory mechanisms, combined treatment strategies have shown synergistic effects. Dual inhibition of MCT1 and MCT4, using compounds likeSyrosingopine, is more effective than targeting a single pathway, both *in vivo* and *in vitro*^43^^,^^46^. Additionally, combining LDHA inhibitors with oxidative phosphorylation inhibitors, such as GNE-140 with metformin, effectively blocks energy compensation^119^^,^^120^. The combination of NHE1 and CA inhibitors can block H^+^ excretion and HCO_3_^-^ regeneration due to the metabolic complex formed between NHE1 and CA IX, thereby neutralising the acidic TME and restoring T cell function^64^. Furthermore, using pH regulatory inhibitors alongside immune checkpoint inhibitors can enhance the effectiveness of anti-PD-1/PD-L1 therapies^65^^,^^118^. Future preclinical research and clinical trials should focus on rational combination treatment regimens rather than single-agent interventions. It is crucial to improve the subtype selectivity of inhibitors and reduce off-target toxicity through precise molecular design and protein degradation technologies. Additionally, developing pH-responsive delivery systems and acidic targeting strategies, optimising hydrazone bond linkers, and constructing pH-sensitive lipid-nanoparticle composite systems should be pursued to enhance drug accumulation at tumour sites^121–125^. As mechanistic understanding deepens and technological innovations advance, strategies targeting the acidic microenvironment of tumours will move from basic research to clinical application, offering a new paradigm of high efficacy and low toxicity for treating solid tumours.

## Data Availability

Data sharing is not applicable to this article as no new data were created or analysed in this study.
